# Noncytotoxic silver nanoparticles as a new antimicrobial strategy

**DOI:** 10.1038/s41598-021-92812-w

**Published:** 2021-06-29

**Authors:** Bartosz Skóra, Urszula Krajewska, Anna Nowak, Andrzej Dziedzic, Adriana Barylyak, Małgorzata Kus-Liśkiewicz

**Affiliations:** 1 Department of Biotechnology and Cell Biology, Medical College, University of Information Technology and Management, St. Sucharskiego 2, 35-225 Rzeszów, Poland; 2 Institute of Biology and Biotechnology, College of Natural Sciences, University of Rzeszow, St. Pigonia 1, 35-310 Rzeszów, Poland; 3 College of Natural Sciences, University of Rzeszow, St. Pigonia 1, 35-310 Rzeszow, Poland; 4 Laser Department Center of Imlantation and Prosthetic Dentistry “MM”, Department of Therapeutical Dentistry, Lviv National Medical University Ukraine, Lviv, Poland

**Keywords:** Biotechnology, Microbiology

## Abstract

Drug-resistance of bacteria is an ongoing problem in hospital treatment. The main mechanism of bacterial virulency in human infections is based on their adhesion ability and biofilm formation. Many approaches have been invented to overcome this problem, i.e. treatment with antibacterial biomolecules, which have some limitations e.g. enzymatic degradation and short shelf stability. Silver nanoparticles (AgNPs) may be alternative to these strategies due to their unique and high antibacterial properties. Herein, we report on yeast *Saccharomyces cerevisiae* extracellular-based synthesis of AgNPs. Transmission electron microscopy (TEM) revealed the morphology and structure of the metallic nanoparticles, which showed a uniform distribution and good colloid stability, measured by hydrodynamic light scattering (DLS). The energy dispersive X-ray spectroscopy (EDS) of NPs confirms the presence of silver and showed that sulfur-rich compounds act as a capping agent being adsorbed on the surface of AgNPs. Antimicrobial tests showed that AgNPs inhibit the bacteria growth, while have no impact on fungi growth. Moreover, tested NPs was characterized by high inhibitory potential of bacteria biofilm formation but also eradication of established biofilms. The cytotoxic effect of the NPs on four mammalian normal and cancer cell lines was tested through the metabolic activity, cell viability and wound-healing assays. Last, but not least, ability to deep penetration of the silver colloid to the root canal was imaged by scanning electron microscopy (SEM) to show its potential as the material for root-end filling.

## Introduction

Silver nanoparticles (AgNPs), due to their unique chemical, physical and biological properties, are one of the most commonly tested nanoparticles in nanobiotechnology^1,2^. Various routes, including physical, chemical and biological procedures, have been developed to synthesize silver nanoparticles. However, an increased demand in eco-friendly processes and using biocompatible reagents make a great of interest the biological way of synthesis of nanoparticle^3^. During the green synthesis, biological compounds from bacteria, microalga and yeasts are used, instead of chemical formulas, which makes the reaction more environmentally friendly. Among many organisms, *Saccharomyces cerevisiae*, nonpathogenic and non-toxigenic, generally recognized as safe (GRAS) organism should be considered as a promising “tool” for green silver nanoparticles synthesis. Indeed, Niknejad et al. demonstrated the potential of *S. cerevisiae* for extracellular synthesis of fairly monodisperse silver nanoparticles and proved their antifungal activity^4^. Furthermore, obtaining the functionalization of biogenic silver nanoparticles with a biocompatible compound, came from yeast extract, would further enhance their biological activities^3^. Moreover, multi-sites action of nanosilver could be a promising candidate to overcome the ongoing microbial resistance^4^.

Microbial adhesion, resulting in biofilm formation, is very often attributed or associated to the number of diseases. It is a significant virulence mechanism in the pathogenesis of many medically important bacterial pathogens^5^. Unfortunately, most currently used antimicrobial treatments are developed against planktonic bacteria^6^. Moreover, biofilms are 10- to 1000-fold more adaptively resistant to antimicrobials than planktonic bacteria, probably because the biofilm antibiotic tolerance is thought to involve alternative mechanisms to bacterial antimicrobial resistance^5,7^. According to the US Centers for Disease Control, 80% of human bacterial infections are due to biofilms, and therefore, they pose a significant problem to human health^7,8^. Thus, due to the failure in the prevention or eradication of microbial biofilm, it is a great demand to look for a new strategy and treatment.

Up to date, there are a few approaches to comb with recalcitrant biofilms; i.e. electrochemical treatment, use the antimicrobial compounds or biomolecules which exhibit antibiofilm activity and target the biofilm architecture, as well as drug delivery methods [review in^7^. In the latter one, especially engineered nanoparticles have been explored as drug delivery vehicles, where compound of interest is adsorbed to a nanomatrix or entrapped by it^9^. However, targeting of bacteria pathogens with nanocarriers may have some limitation, such as shelf life, stability, encapsulation efficacy, drug release/leakage, and enzymatic degradation of loaded compound^10^. To overcome these disadvantages, silver nanoparticles with its antimicrobial efficacy, may become an alternative strategy. Indeed, these nanostructure have been used to control bacterial colonization and infection in wound healing^11^. Moreover, some authors demonstrated that AgNPs can interact with mature bacterial biofilm, and showed their high toxicity against pathogens^12^. The strong antimicrobial effect of silver nanoparticles is indisputable. However, when they are supposed to be applied to humans, their toxic effect to eukaryotic cells must be considered. The cytotoxicity of AgNPs was noticed for human cervical cancer cells (HeLa), human lung carcinoma (A549), and human hepatocellular carcinoma (Hep-G2)^13–15^. Unfortunately, there is still gap in knowledge on the impact of the silver nanoparticles toward human cells when its antibacterial potential is taken into account the human application. For example, the lack of the cytotoxicity of silver nanoparticles toward eukaryotic cells, with their antibacterial potential may be very useful for endodontic treatment when the elimination of bacteria in the root canal system is required^16,17^. Pathogenic microorganisms are able to penetrate the root dentin up to a depth of more than 1 mm, whereas disinfecting solutions only reach a depth around 100 µm^18,19^. Dentin with its structural features as dentinal tubules, which are filled with pathogenic germs in dentinal tubules is an ideal object for disinfection of nanoparticles. Thus, the new devices/compounds which may help to overcome the problem of the insufficient penetration depth into the tooth are very desirable.

The aim of the study was to describe the antibacterial effect of the green-synthetized silver nanoparticles, simultaneously with the analysis of their cytotoxic effect on eukaryotic cells. The antimicrobial potential was checked through either the ability to inhibit the formation of biofilm or to eradicate already existed one, for *Pseudomonas aeruginosa* and *Escherichia coli*. Moreover, the growth was monitored also through the zone of inhibition assay for *Staphylococcus aureus* and *Candida albicans* strains. To assess the cytotoxicity, the impact of AgNPs on metabolism activity, viability and migration ability of mammalian cell lines (mouse embryonic fibroblasts, human keratinocytes, human osteosarcoma and human non-small cell lung carcinoma) was demonstrated. Bioformation of silver nanoparticles was monitored by UV–Vis spectroscopy. The structure and chemical composition of silver nanoparticles were evaluated with electron transmission microscopy imaging. The size and stability of the nanostructures were analyzed by hydrodynamic light scattering measurements. Moreover, the ability of the silver nanoparticles to penetrate the root canal has been imaging.

## Material and methods

### Reagents

Yeast extract Peptone Dextrose medium (YPD), Luria–Bertani (LB) and agar were purchased from BTL (Poland). Acridine orange (AO), 1-(4,5-Dimethylthiazol-2-yl)-3,5-diphenylformazan (MTT), crystal violet (CV), ethanol were purchased from Sigma-Aldrich (USA). Dimethyl sulfoxide (DMSO) and ethidium bromide (EtBr) were purchased from Chempur and MpBio, respectively. Dulbecco's Modified Eagle Medium High Glucose (DMEM, Corning), antibiotic solution (Antibiotic Antimycotic Solution, Sigma-Aldrich), fetal bovine serum (FBS, Biowest), phosphate buffered saline w/o magnesium and calcium (PBS, Corning), 0.25% trypsin (PAA) were used.

### Microorganisms and cell lines

*Saccharomyces cerevisiae* (10,058/69 strain) were previously isolated from patient mouth, delivered by Department of Microbiology (Medical University of Wrocław) and genetically identified^20^. The YPD medium (10 g/L yeast extract, 10 g/L peptone, 20 g/L glucose) was used for preculture and the yeasts were grown in flat-bottom flask at 28 °C with rotatory shaking (150 rpm). For antimicrobial assays, bacteria (*Staphylococcus aureus* ATCC 25,923, *Pseudomonas aeruginosa* ATCC 27,853, *Escherichia coli* PCM 2209) and fungi (*Candida albicans* ATCC 14,053) strains were employed.

Bacteria were precultured in LB medium at 37 °C with overnight shaking (150 rpm), while fungi in YPD medium at 30 °C. The cell lines used in this study were mouse embryonic fibroblasts (NIH 3T3, ATCC CRL-1658), human keratinocytes (HaCaT, ATCC CRL-2522), human osteosarcoma (U-2OS, ATCC HTB-96) and human non-small cell lung carcinoma (NCI-H1299, ATCC CRL-5803). They were grown and handled according to standard technique as described elsewhere^21^. Cultures were performed in 24- or 96-well plate (Corning) containing 1.5 mL and 200 µL of DMEM medium for wound-healing assay and MTT assay or AO/EB staining, respectively. Medium was supplemented with 1% of antibiotics solution and 10% of fetal bovine serum and cultures were incubated at 37 °C in a 5% CO_2_ air saturated incubators. 24 h before the experiments, cells were seeded at a density 5 × 10^4^ and 5 × 10^3^ cells/well for 24- or 96-well plate, respectively.

### Synthesis of AgNPs

The *Saccharomyces cerevisiae* 10,058/69 strain was used for AgNPs synthesis due to their silver nitrate reduction properties. The production of AgNPs were performed with lower and higher concentration of AgNO_3_, 1 mM and 3 mM (for further analysis the abbreviations were as followed: AgNPs_L or AgNPs_H). Briefly, after 48 h of yeast culturing at optimal temperature 28 °C with shaking, the cell suspension, when reached the density atOD_600_ ~ 14, was centrifuged. The obtained supernatant was removed, followed the yeast biomass was resuspended in sterile distilled water and cultured for next 48 h in 28 °C with shaking. The post-culturing water was collected by centrifugation and its pH was adjusted to 10. Consequently, the silver nitrate was added to the post-culturing water in final concentration 1 mM and 3 mM and placed into 60 °C for 24 h. The specific conditions during the synthesis (i.e. silver ions concentrations, time and pH of the synthesis) were chosen based on our preliminary results (data not shown). For the evaluation of the ability to penetrate the root canal of the tooth, the colloid of silver nanoparticles was developed in collaboration with the Institute of Cell Biology of NAS, Lviv, Ukraine.

### Physicochemical characterization of AgNPs

UV–Vis spectrum of AgNPs was recorded on TECAN spectrofluorometer (Infinite M200, Thermo Scientific) in the range of 330–900 nm. To determine the concentration of AgNPs, the microbalance technique using Radwag MYA 5.4Y balance was used. Briefly, 100 μL of the colloid suspension was placing on aluminum crucible with known mass and evaporating the solvent to a dry mass. Concentration values are given as mean and standard deviation of triplicate. The Dynamic Light Scattering (DLS) and the polydispersity index (PdI) were determined using the universal Nanoplus HD3 system (Particulate System/Micrometrics) equipped with 660 nm laser diode, as described elsewhere^22^. All the analysis were performed at 25 °C. Prior measurement, solutions of AgNPs were sonicated (310 W, 50 Hz, 100%, 10 min, Polsonic, SONIC-3). AgNPs were imaged with transmission electron microscopy (FEI Tecnai Osiris as described elsewhere^23^.

### Antimicrobial potential of AgNPs

The antimicrobial of synthesized AgNPs activity through the zone of inhibition was tested against different pathogens such as *S. aureus*, *P. aeruginosa*, *E. coli* and *C. albicans*, according to a modified standard protocol^24^. Briefly, this modification consisted in punching 5-mm diameter wells in the nutrient agar instead of using a soaked disc. 100 µL of the overnight culture of the strains suspensions were spread onto the agar plates. When dried, 50 µL of AgNPs_1 and AgNPs_3 were aseptically transferred into separate wells. The plates were incubated at 30 or 37 °C for 24 h, for yeast and bacteria respectively. The inhibition zones surrounding the wells were recognized as the ability of the NPs to inhibit the growth of cells. For further analysis, *P. aeruginosa* and *E. coli* strains have been chosen to evaluate the influence of AgNPs on the bacteria ability to create biofilm in the presence of nanoparticles. Here, two type of assays have been implemented, to determine if the AgNPs can (i) prevent biofilm formation or (ii) reduce the final biofilm biomass^25^. (i) To evaluate the inhibition of biofilm formation the overnight bacteria inoculums (~ 10^8^ CFU) were mixed with the colloid of AgNPs in the range of concentrations of 0.125–2 mg/mL. Cells were then incubated for 24 h at 37 °C without agitation, to allow biofilm formation. Plates were washed thrice with PBS from non-adhered cells and dried for 15 min at room temperature. Biofilms were stained for 15 min by adding 0.4% of crystal violet (CV) solution to each tested well. Then after, cells were washed with PBS to remove excess of the dye. Finally, the crystals were solubilized with 30% acetic acid, followed by absorbance measurement at 595 nm using a microplate readers (Infinite M200, Thermo Scientific). Results are reported relative to untreated biofilm biomass, as an OD values. (ii) Degradation of pre-established biofilm was tested as described above with some alterations. Cells were seeded in 96-well plate in LB without adding AgNPs and incubated at 37 °C for 24 h to allow biofilm creating. After this time, the wells were rinsed and replaced with fresh one, containing different concentrations of AgNPs. After 24 h the CV staining as described above was performed. The results were expressed as a percentage of biofilm degradation compare to non-treated cells.

### Cytotoxicity of AgNPs

#### Cell metabolic activity

The 1-(4,5-Dimethylthiazol-2-yl)-3,5-diphenylformazan (MTT) assay was used as an indicator of metabolism activity level of the cell after AgNPs solutions treatment. The assay was performed according to^23^. Briefly, cells were seeded on 96-well plate at density 5 × 10^3^ cells/well, 24 h before the experiment. After this time, the medium was removed and replaced with the fresh one, containing different concentrations of AgNPs colloid (1 mM and 3 mM of precursor variants) in the range of 0.125–2 mg/mL. Cells were cultured for 24 h at 37 °C in a humidified incubator in a 5% of CO_2_ atmosphere. The medium was discarded from each well and 200 µL of fresh DMEM with MTT (0,5 mg/mL) was added into well. After an additional incubation of 2–3 h, to allow the formazans to form, 100 µL of DMSO were added per well to stop the reaction and dissolve crystals. The absorbance was finally measured at 565 nm wavelength on microplate reader (Tecan, Switzerland) and the metabolic activity results were expressed as a percentage of the control (untreated cells). All tests were performed at least in triplicate.

#### Cell viability

Examination of cell viability has been conducted by ethidium bromide (EB) and acridine orange (AO) staining to distinguish live and death cells, according to^26^. Briefly, confluent cells were incubated for 24 h in the presence of 0.5 or 1 mg/mL of AgNPs. After this time, plates were centrifuged (300 × g, 5 min) and supernatant was discarded. Cells were washed with PBS and were stained with a solution of acridine orange (100 μg/mL in PBS) and ethidium bromide (100 μg/mL in PBS) at a volume ratio of 1:1 for 5 min. Under fluorescence microscope (Olympus, Japan) living cells were visualized as green while dead cell were stained in red. For cell viability determination, a total of 100 cells from image (at least six images from different wells were examined) were counted and dead cells were expressed as a percentage of the total number of the cells.

#### Wound-healing assay

The impact of AgNPs on cells migration were performed using wound-healing assay (scratch assay), according to^27^. Briefly, the cells were seeded at a density 5 × 10^4^ cells/well in 24-well plate and incubated until reached 90% of confluence. Wells with confluent cells were scratched by using a P10 pipette tip in the diameter of the culture. Then after, the medium was removed, and the cells were washed with PBS to discard detached cells. Followed, the 0.5 mg/mL of AgNPs solution suspended in DMEM with 1% of FBS was added to each well and cultured for 24 and 48 h. Wound closures were periodically visualized (0, 24 and 48 h intervals) under inverted microscope. The ability of cell to migrate and wound closure after AgNPs treatment was calculated with using ImageJ software according to^28^. The results were expressed as a percentage of the scratch closure compared to scratch surface at 0 h time in each group. All tests were performed at least in duplicate.

### Electron scanning microscopy of the root canal penetration

To examine the tooth samples in an electron scanning microscope, 44 endodontically developed.

single-rooted human teeth with straight canals were taken, in which 2 μL of a solution of silver nanoparticles were injected. The tooth samples were prepared in the longitudinal direction of the root canal to visualize the lumen of the dentinal tubules. The study was performed using an electron scanning microscope (ESEM XL30, Philips, Netherlands).

### Statistical analysis

The results represent the mean ± SD from at least three independent experiments. One-way ANOVA with Tuckey post-hox test, using Graph 10 software (*P < 0.05; **P < 0.01 and ***P < 0.001) was performed (*P < 0.05; **P < 0.01 and ***P < 0.001) compare to the control group. t-test was also used to determine statistical difference between scratch closure (HaCaT vs. U-2OS) (#*P* < 0.05).

## Results and discussion

### Physicochemical characterization of AgNPs

**Figure 1:**
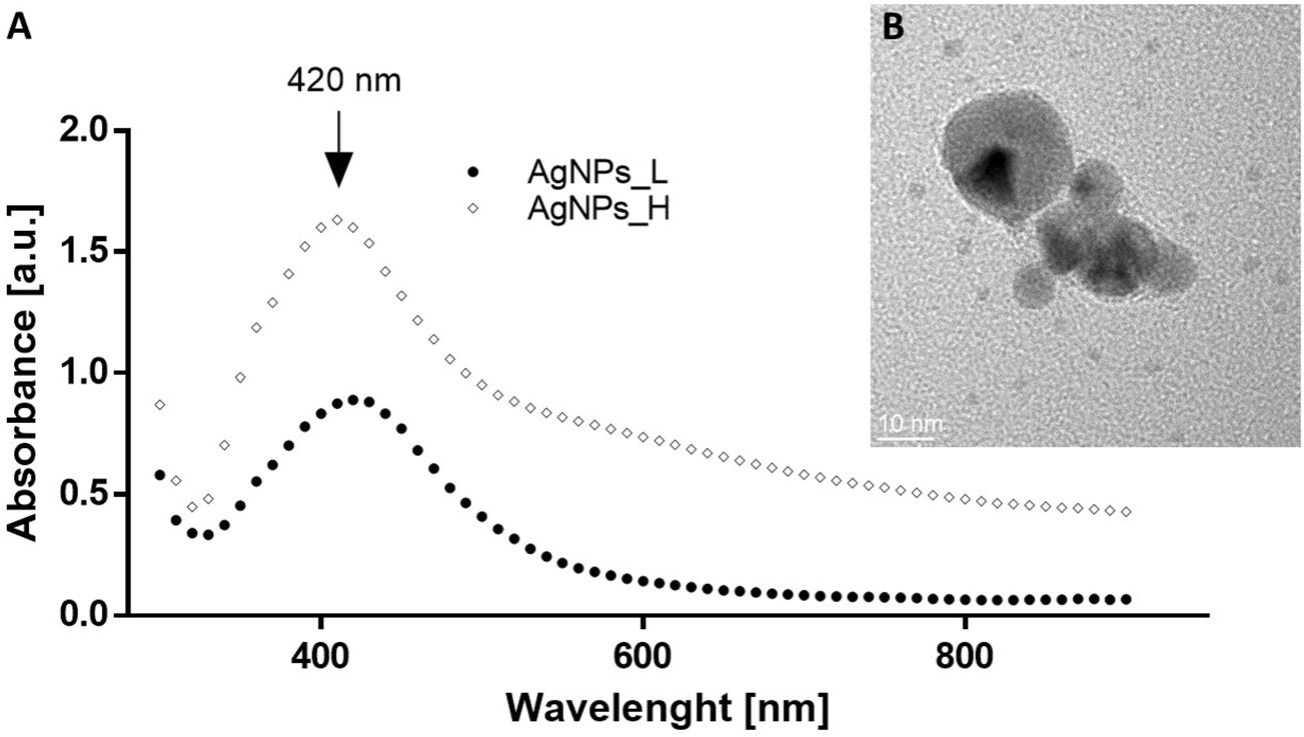
(**A**) UV–Vis absorption spectra of biosynthesized AgNPs_L (circle) and AgNPs_H (diamond). (**B**) Example of TEM image of AgNPs.

The ability of yeast water extract to reduce silver ions in the reacting solution and formed the silver nanoparticles was monitored with UV–visible spectra, where specific surface plasmon resonance (SPR) should appear during nanoparticles formation^29^. SPR is the absorption of the visible electromagnetic radiation of the collective oscillations of surface electrons^30^. Indeed, the maximum absorption peak was observed at 420 nm, for both of the samples (Fig. 1A). This wavelength was identical as reported by Elamawi et al., who obtained silver NPs synthetized from the cell-free fungal extract of *Trichoderma* sp.^31^. And was also closely matched (410 nm) to those obtained from the cell-free filtrate of *Aspergillus fumigatus*^32^. According to the Mie’s theory only a single SPR band is expected in the absorption spectra of spherical metal nanoparticles^33^. We present that silver nanoparticles absorb blue light and exhibit one single peak, thus, they are spherical in shape. Moreover, morphology of the synthesized NPs was confirmed with transmission electron microscopy (Fig. 1B), where predominantly spherical shape was imaged.

**Figure 2:**
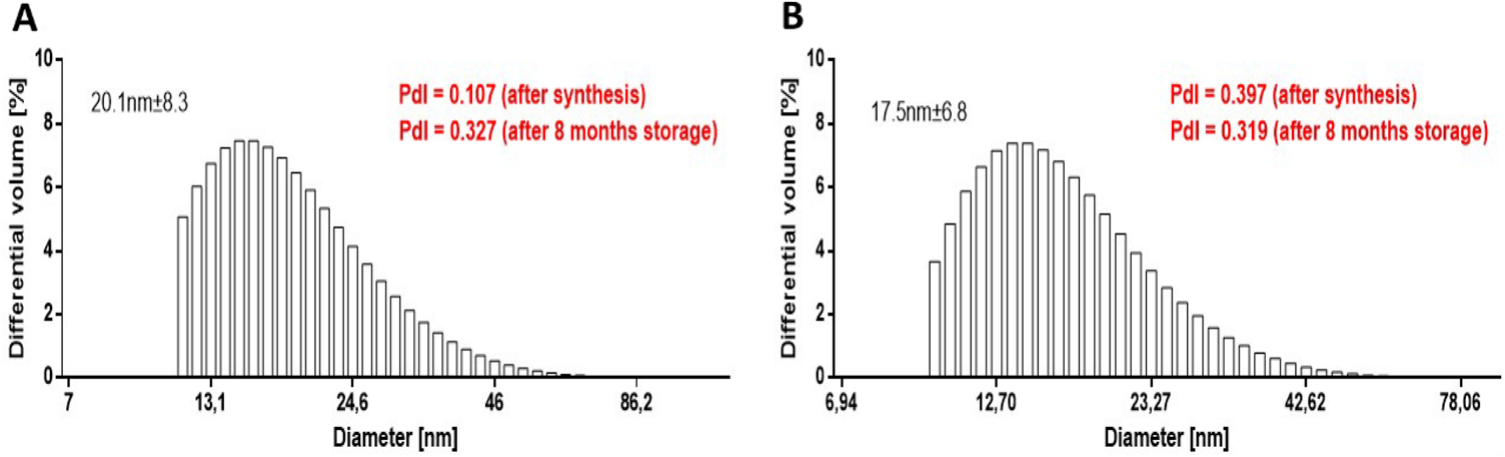
Size of the AgNPs_L (**A**) and AgNPs_H (**B**) evaluated by hydrodynamic light scattering analysis. PdI, polidyspersity index.

For various bioapplications, physicochemical properties determine nanomaterial cellular uptake, transport and fate^34^. Considering this, here we evaluated some of the most important features of the nanomaterials, primarily the size, stability and surface chemistry of biomanufactured AgNPs. The dynamic light scattering (DLS) was used to study the size distribution and colloidal stability of AgNPs^35^. The synthetized silver nanoparticles presented a size with median value 20.1 nm and 17.5 nm, for the sample AgNPs_L and AgNPs_H, respectively (Fig. 2). The stability of the nanoparticles as colloid is very important, as unstable NPs will not be able to disperse homogenously, which may effect on their antibacterial properties and reducing the efficacy^31,36^. Therefore, the polidyspersity index (PdI) was used as a value which show the stability of the nanomaterial. The higher the PdI value is, the less monodispersed are the nanoparticles^37^. In this study, the PdI of the materials were equal to 0.107 and 0.397 after synthesis or 0.327 and 0.319, after 8 month storage, for the AgNPs_L and AgNPs_H, respectively (Fig. 2). Thus, this suggest the nanoparticles are stable as they do not exhibit any considerable aggregation. It is worth to mention that many papers reported on difficulties in the synthesis of stable solution of NPs due to their tendency to agglomerate^38–40^. According to Gorham et al. PdI of AgNPs increased due to oxidation-dependent processes. The authors reported that citrate-coated AgNPs are characterized by increasing of agglomeration level during 104-day storage, despite citrate use^41^. Similar effect was observed by Izak-Nau et al., who show that agglomeration of citrate-coated AgNPs can be delayed effectively about 6 months since being synthesized. After this time, the hydrodiameter size of NPs in tested solutions increased significantly^42^. Interestingly, our results clearly showed that using post-harvested yeast water during synthesis allow to obtain stable NPs solutions, as PdI is not increased significantly, even after 8-month-storage.

**Figure 3:**
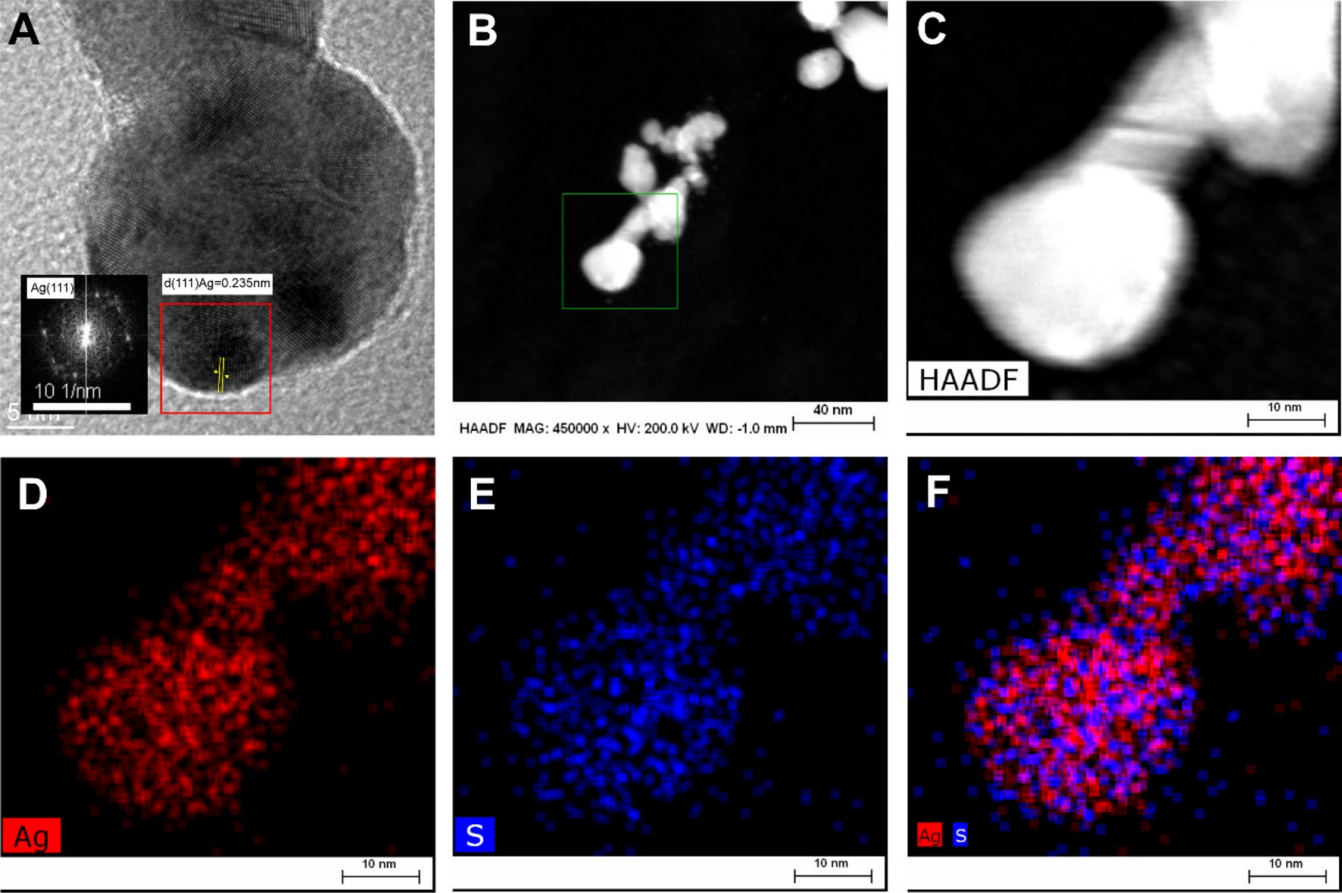
(**A**) Representative HRTEM images of single silver nanoparticle with lattice fringes. Silver was identified by the inter-planes spacing d = 0.235 nm corresponding to the (111) plane of silver. Measurement of inter-planes spacing was done on FFT image. (**B**, **C**) HAADF STEM image of AgNPs with various magnification. (**D**–**F**) The chemical composition analysis of AgNPs. EDS elemental mapping images of silver (red), sulfur (blue), and an overlay of the Ag and S. All the EDS measurements are collected from image B (lower magn.) and C (higher magn).

The morphology and elemental composition of the AgNPs were determined by transmission electron microscopy (TEM) and energy dispersive X-ray spectroscopy (EDS). Figure 3A depicts the HRTEM image of biosynthesized silver nanoparticles showing the lattice fringes clearly. The calculated inter planar distance was equal to 0.235 nm, confirmed occurrence of phase of Ag. According to the STEM-HAADF and EDS results (Fig. 3B-F) the synthesized nanomaterial is mainly constituted of Ag (Fig. 3D). Interestingly, the presence of sulfur precisely covering the nanoparticles was mapped (Fig. 3E). This may suggest that biocompounds reach in sulfur, which exists in the yeast water extract, may have the capping and stabilizing role. Many studies have revealed that the use of inorganic stabilizers such as citrate, PVP or PAA during synthesis allow to obtain stable NPs solutions^43,44^. However, some of them can influence negatively on human health e.g. PAA, which may cause the irritation of respiratory system after inhalation^45^. On the other hand, during the biological synthesis of nanoparticles, some biocompounds i.e. exopolysaccharides or proteins may exist as a stabilizing agent when nanoparticles are formed^46,47^. The sulfur which was imaged on Fig. 3E suggests the presence of some yeast proteins coating the surface of NPs. According to the above-mentioned literature, we suppose that during the synthesis, the *Saccharomyces cerevisiae* proteins or sulfur-rich biocompund coating the AgNPs surface and stabilize them, allowing to maintain stable while storage.

### Antimicrobial effect of AgNPs

#### Zone of inhibition

**Figure 4:**
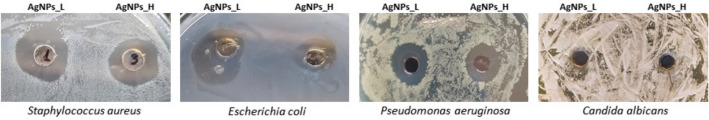
Antimicrobial activity of the AgNPs after 24 h of incubation against *Staphylococcus aureus*, *Escherichia coli*, *Pseudomonas aeruginosa* and *Candida albicans*.

Antimicrobial potential of the silver nanoparticles is ascribed to their diverse mechanism of action, and it is believed as the multistep and multilevel process^48^. To evaluate this potential, the biosynthesized AgNPs (both of the tested variants) were analyzed against most pathogenic strains of bacteria: *Escherichia coli*, *Pseudomonas aeruginosa* and *Staphylococcus aureus* and fungi *Candida albicans*, through the zone of inhibition assay. After 24 h of exposure, the growth was inhibited for each of the bacteria strains, whereas no inhibition was observed for yeast strain for both of the tested samples (Fig. 4). Kota et al. showed that AgNPs were characterized by high antibacterial properties, confirmed by analysis on sixteen pathological isolates from human, both Gram positive and Gram negative strains^49^. Moreover, the authors proved that 50 µg/µL concentration of these nanoparticles were able to increase zone of bacteria growth inhibition with the same efficiency in all tested isolates^49^. Interestingly, Jalal et al. showed high antifungal properties of AgNPs (extracellularly synthetized by *C. glabrata* supernatant) towards six *Candida species*^50^. Similarly results were presented by Perween et al., who reported on potential usefulness of AgNPs in *C. albicans* infections, better than well-known antifungal xenobiotics^51^. These conclusions are in the opposition of our results, which showed no effect on *C. albicans* growth inhibition of tested AgNPs solutions, which may be caused by higher diameter of tested AgNPs and/or sulfur coating of NPs surface.

#### Inhibition of biofilm formation

**Figure 5:**
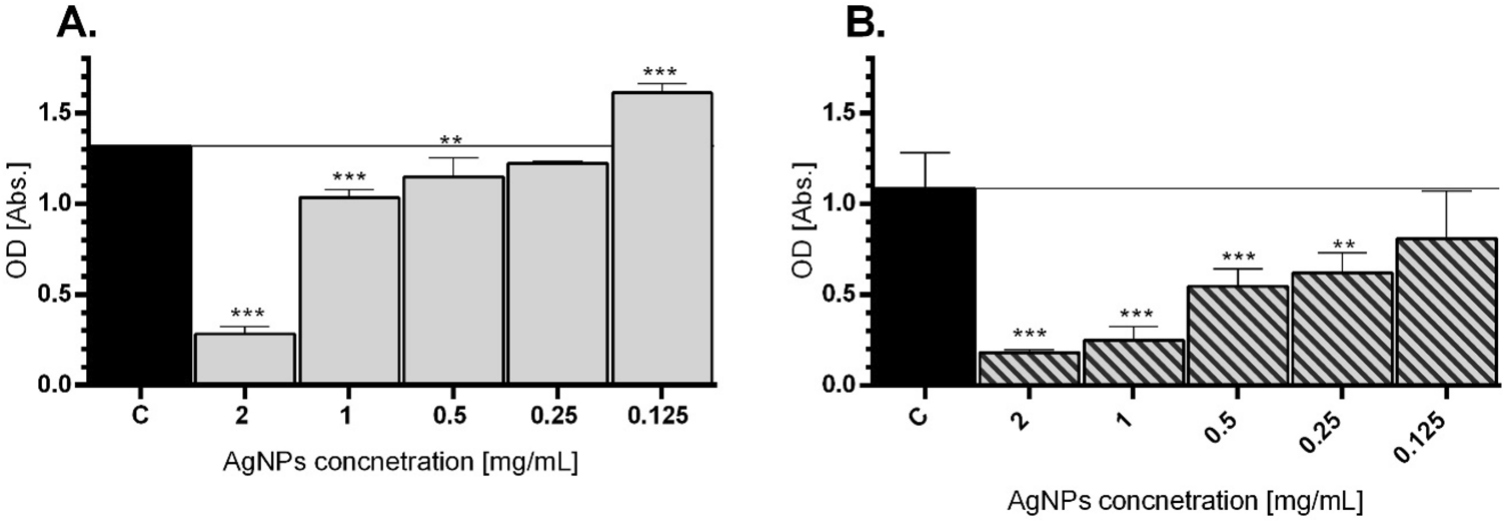
Biofilm inhibition after treatment of AgNPs in *E. coli* (**A**) and *P. aeruginosa* (**B**). Strains were incubated for 24 h in the presence of AgNPs. Post-treatment surface-associated biofilm was stained and the OD of biofilm biomass were presented. Mean values with standard deviation (error bars) with *, **, ***are statistically different from the respective control at *P* < 0.05, *P* < 0.01, and *P* < 0.001, respectively (one-way ANOVA, Tukey test).

Bacterial colonization on abiotic or biotic surfaces may leads to biofilm formation and these microbial aggregates in biofilms produce a blockade that resists antimicrobial agents^48^. Thus, due to extremely small sizes of NPs, they may be useful for accomplishing antimicrobial actions and fighting intracellularly with bacteria^52^. Herein, the antibiofilm efficacy of the silver nanoparticles was evaluated with the crystal violet staining assay, in a case of ability to inhibit biofilm formation. Different biofilm percentage of reduction was detected for inhibition biofilms when treated with different concentrations of AgNPs_L, however, in the concentration dependent manner. For the *E. coli* strain, the best reduction was achieved at the highest concentration 2 mg/mL, which causes reducing the OD biofilm from 1.3182 (control) to 0.2806 in the tested group (Fig. 5A). While *P. aeruginosa* exhibits decreasing of biofilm OD up to 0.1813 (control group 1.0831) after 24 h treatment of 2 mg/mL concentration of AgNPs_L (Fig. 5B). Various mechanisms of antibacterial properties of AgNPs are described in the literature. Among them the high level of ROS production and the failure to eliminate them by *P. aeruginosa* after silver NPs exposition was noticed^53^. Thus, it is suggest that AgNPs may become an antimicrobial agent on the multidrug-resistant strain, which is an ongoing problem in the medicine^53^. Similarly, our results showed the high antibacterial potential of AgNPs_L against *E. coli* and *P. aeruginosa.* Moreover, the presented results revealed the potential of tested AgNPs to prevent the creation of bacterial biofilm. Masurkar et al. highlighted that AgNPs were able efficiently to inhibit biofilm of *Staphylococcus aureus*, comparable to antibiotic-treated group^54^. Martinez-Gutierrez et al. presented a comprehensive report about negative impact of AgNPs on many clinically important bacteria strains i.e. *S. aureus*, *S. epidermidis* and *A. baumanni* which are considered to be problematic in hospital treatment^55^. Our results clearly showed that AgNPs synthetized with easy, cheap, fast and cost-effective way, may have high application value to treat pathogenic strains.

#### Biofilm eradication

**Figure 6:**
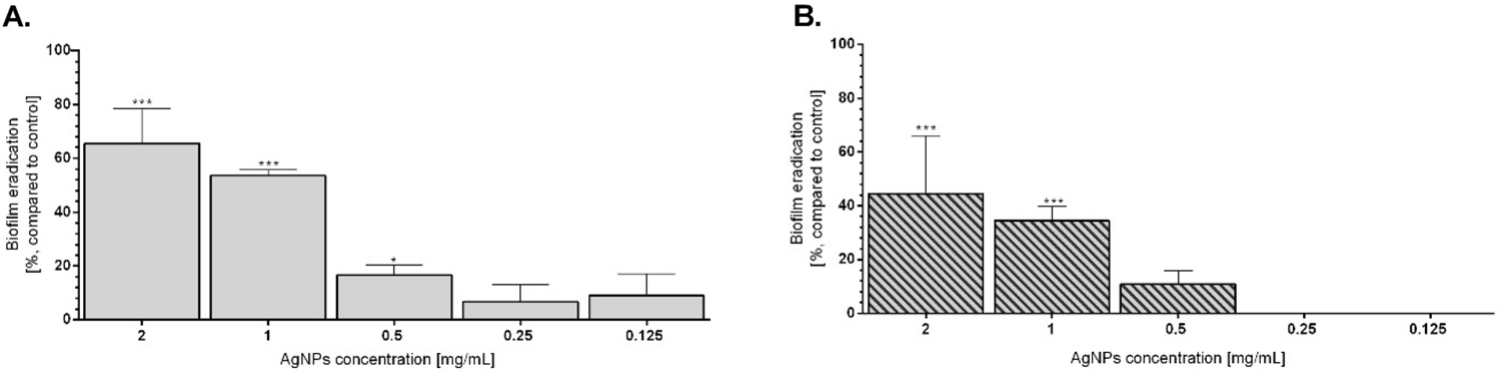
Biofilm eradication after treatment of AgNPs in *E. coli* (**A**) and *P. aeruginosa* (**B**). Created biofilms were treated with AgNPs for 24 h and the percentage of biofilm eradication in comparison to untreated bacteria was calculated. Mean values with standard deviation (error bars) with *, **, ***are statistically different from the respective control at *P* < 0.05, *P* < 0.01, and *P* < 0.001, respectively (one-way ANOVA, Tukey test).

The presence of bacterial biofilms is an emerging problem in nowadays hospital infections, due to high resistance of these structures on antibiotic^56,57^. Many xenobiotics were tested as a potential anti-biofilm agents, however the highest efficiency of biofilm eradication was proved by AgNPs^58,59^**.** Therefore, herein the efficiency of AgNPs ability to *E. coli* and *P. aeruginosa* biofilm eradication was evaluated (Fig. 6). In both tested bacterial strains, AgNPs caused decreasing in biofilm biomass. The highest eradication was observed for 1 mg/mL and 2 mg/mL concentrations for both tested strains and reached 65% and 53% in *E. coli* or 44% and 36% in *P. aeruginosa*, respectively (Fig. 6). The degradative effect of AgNPs in dose-dependent manner was observed, providing by higher eradication of established biofilm of 2 mg/mL and 1 mg/mL concentrations in comparison to 0.5 – 0.125 mg/mL concentrations of tested AgNPs (Fig. 6) *E. coli* established biofilm was more sensitive to AgNPs presence than the *P. aeruginosa* one (Fig. 6). Similar antibacterial effect on *E. coli* biofilm was also proved by Goswami et al., who showed the AgNPs (synthetized by tea leaf extract) were able to eradicate biofilm up to 100%, similar properties of this AgNPs were also observed for *S. aureus* biofilm^60^. Moreover, Ching-Yee et all. showed that citrate-coated AgNPs were characterized by high antibacterial properties against *P. aeruginosa* biofilm and caused its detachment^61^. Nevertheless, many literature data show that biofilm eradication ability of AgNPs is strict correlated with concentration – the higher AgNPs content, the more effective biofilm degradation^62,63^. The same tendency was observed in our results, proving that AgNPs usually act in dose-dependent manner and can be useful in treatment of antibiotic-resistant bacterial strains.

### Biological activity

#### Cell metabolic activity and viability

**Figure 7:**
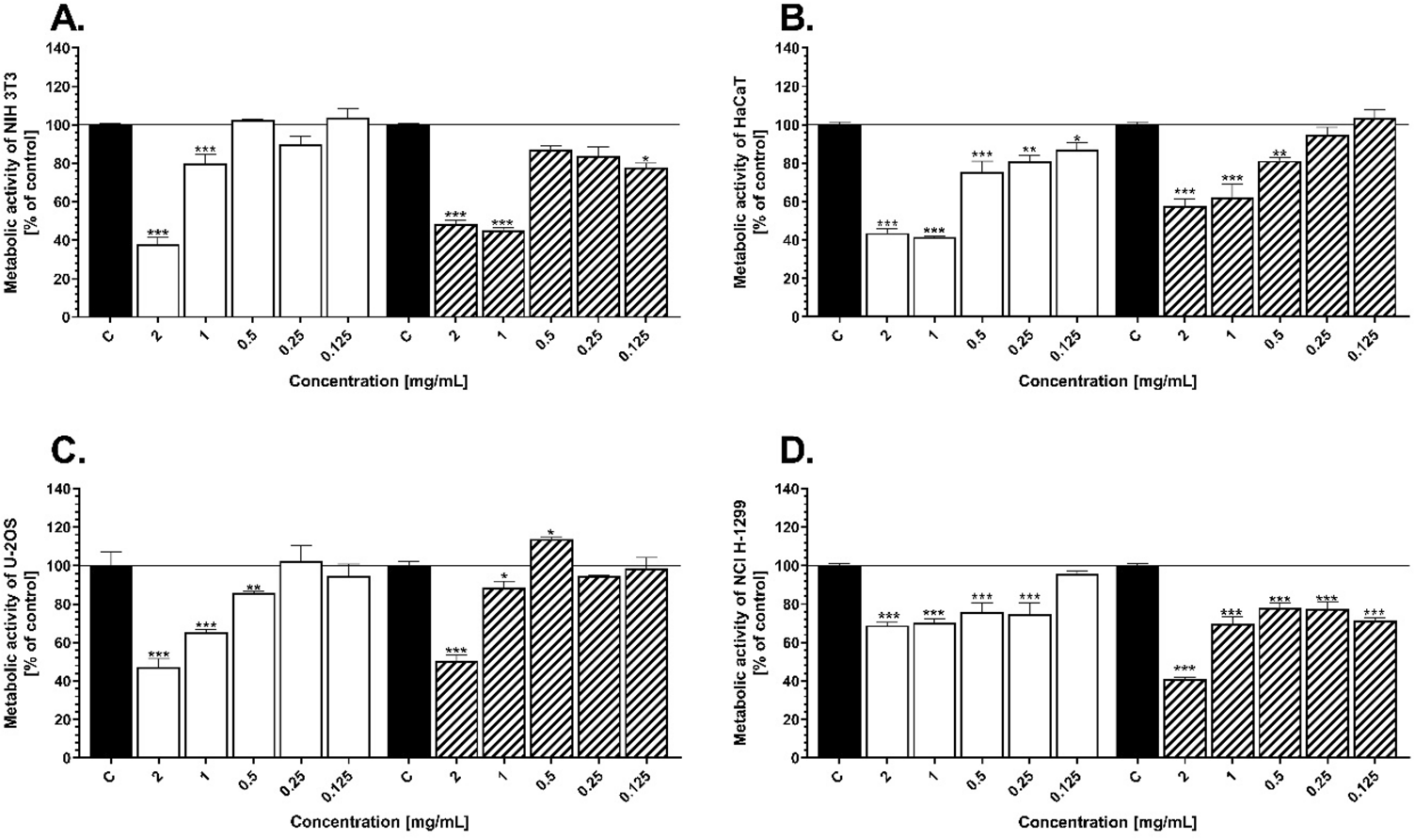
Cell metabolic activity of the NIH 3T3 (**A**), HaCaT (**B**), U-2OS (**C**), and NCI-1299 (**D**) after 24 h exposure to various concentration of AgNPs_L and AgNPs_H. The values are the means (n = 6) with standard deviation (error bars). The statistical significance is indicated as follows: *P < 0.05, **P < 0.01, ***P < 0.005 according to one-way ANOVA, Tukey test.

The potential effect of AgNPs on cell metabolic activity was tested using MTT assay which measures the cell mitochondrial activity through NAD(P)H-dependent cellular oxidoreductase enzyme^64^. The toxicity of various concentrations of the silver nanoparticles (in the range of 0.125–2 mg/mL) toward four different cell lines: mouse embryonic fibroblasts (NIH 3T3), human keratinocytes (HaCaT), human osteosarcoma (U-2OS) and human non-small cell lung carcinoma (NCI-1299) is shown on Fig. 7. Generally, cell metabolic activity was decreased in a dose-dependent manner for human fibroblasts, keratinocytes and osteosarcomas (Fig. 7A-C). While, NCI-1299 cell line exhibits similar level of toxicity no matter on the concentration of the NPs (Fig. 7D). The highest significant (***P < 0.05) decrease was obtained at 2 mg/mL for each tested line, for both type of sample (AgNPs_L and AgNPs_H) compared to the nontreated cells. Comparing the results for cancer and normal cell lines, after their exposure toward two highest concentrations of AgNPs (2 and 1 mg/mL), it is shown that the cancer cells exhibit higher level of metabolic activity in the range from 48 to 73% (compared to control), respectively for U-2OS and NCI H1299 cell lines, contrary to normal cells (HaCaT and NIH 3T3), which metabolic activity was in the range from 37 to 43%, in comparison to control (Fig. 7). It is known that AgNPs are one of the most reactive metal nanoparticles^65,66^. The cytotoxicity effect of these nanostructures is correlated to ROS generation, after uptake inside the cell^2^. According to Kumari et all. cancer cell lines are more resistant to oxidative stress generation, due to their adapting ability^67^. Our results showed higher toxicity of AgNPs in normal keratinocytes and fibroblasts than in cancer ones, which confirm the higher resistance of these cell types to AgNPs-dependent oxidative stress (Fig. 7). On the other hand, Garvey et al. proved higher toxicity in lung carcinoma cells in comparison to normal human keratinocytes. However, the authors used chemically synthetized AgNPs citrate-coated, which are well-known of their high toxicity^68^. Capping agent attached to the surface of nanomaterial may have an impact on their biological activity. Indeed, our EDS results (Fig. 3D-F) showed that silver nanoparticles have been coated with sulfur-rich molecules, which act as a stabilizers. Therefore, we supposed these compounds decrease direct contact of high-reactive AgNPs surface with cells and decrease their toxic effect. This is in line with Senthil et al. who reported on the green synthetized AgNPs and showed less cytotoxicity effect on HaCaT cells, but higher antibacterial properties^69^.

**Figure 8:**
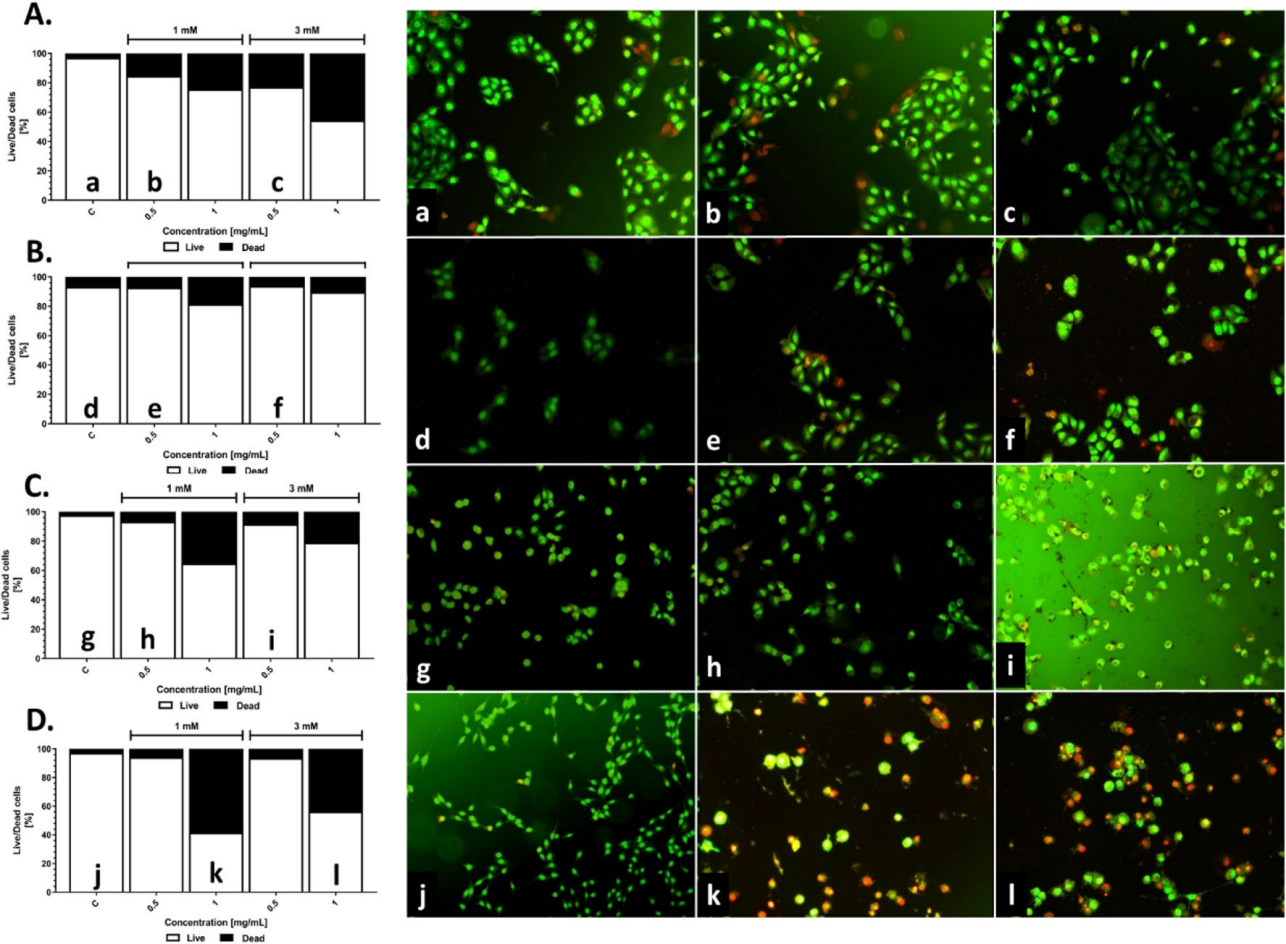
Cell viability of the NIH3T3 (**A**), HaCaT (**B**), U2OS (**C**) and NCI-1299 (**D**) after exposure to AgNPs_L and AgNPs_H. Cells after NPs treatment were stained with acridine orange and ethidium bromide, and were imaged with inverted fluorescence microscope (representative images—right panel). Dead cells were scored per 100 total cells analyzed and expressed as % (graphs).

Many authors usually report on the cytotoxicity of the nanomaterials toward cells based on the only one method used for the analysis^70–74^. Considering only mitochondria activity, it may give the false positive results due to cells exhibit some activity even in early and late apoptosis stadium^75^. Therefore, in this study, either the AO/EB staining or MTT assays have been implemented to evaluate the ratio of the live and dead cells or metabolic activity, respectively. The dual acridine orange/ethidium bromide (AO/EB) staining assay was used to discriminate the live and dead cells after exposure to silver nanoparticles. Confluent cells were incubated with 0.5 and 1 mg/mL of AgNPs_L or AgNPs_H for 24 h and were labeled by AO/EB. For the higher NPs concentration (1 mg/mL), the number of dead cells was significantly increased for each type of cell line (Fig. 8A-D). Contrary, the number of live cells exposure to 0.5 mg/mL of AgNPs was still around 95%. Thus, this confirms the previous results and exhibits no or low toxicity in the range of 0.125–0.5 mg/mL of AgNPs. Representative images of the cells stained with AO/EB are shown on Fig. 8a-l, where red and green colors are dead and live cells, respectively. Based on this result, for further wound healing assay the AgNPs_L sample was used. Sambale et al. used the LDH release level as an indicator of AgNPs toxicity in lung carcinoma (A549) and proved that tested nanostructures did not significantly change the LDH level in the medium, highlighting that cytotoxicity effect of AgNPs is related to stabilizer and cells type^76^. Similarly, our results allow to concluded that the tested nanostructures (in the lower concentration of NPs) were more toxic for normal cell lines than for cancer ones.

#### Wound healing assay

**Figure 9:**
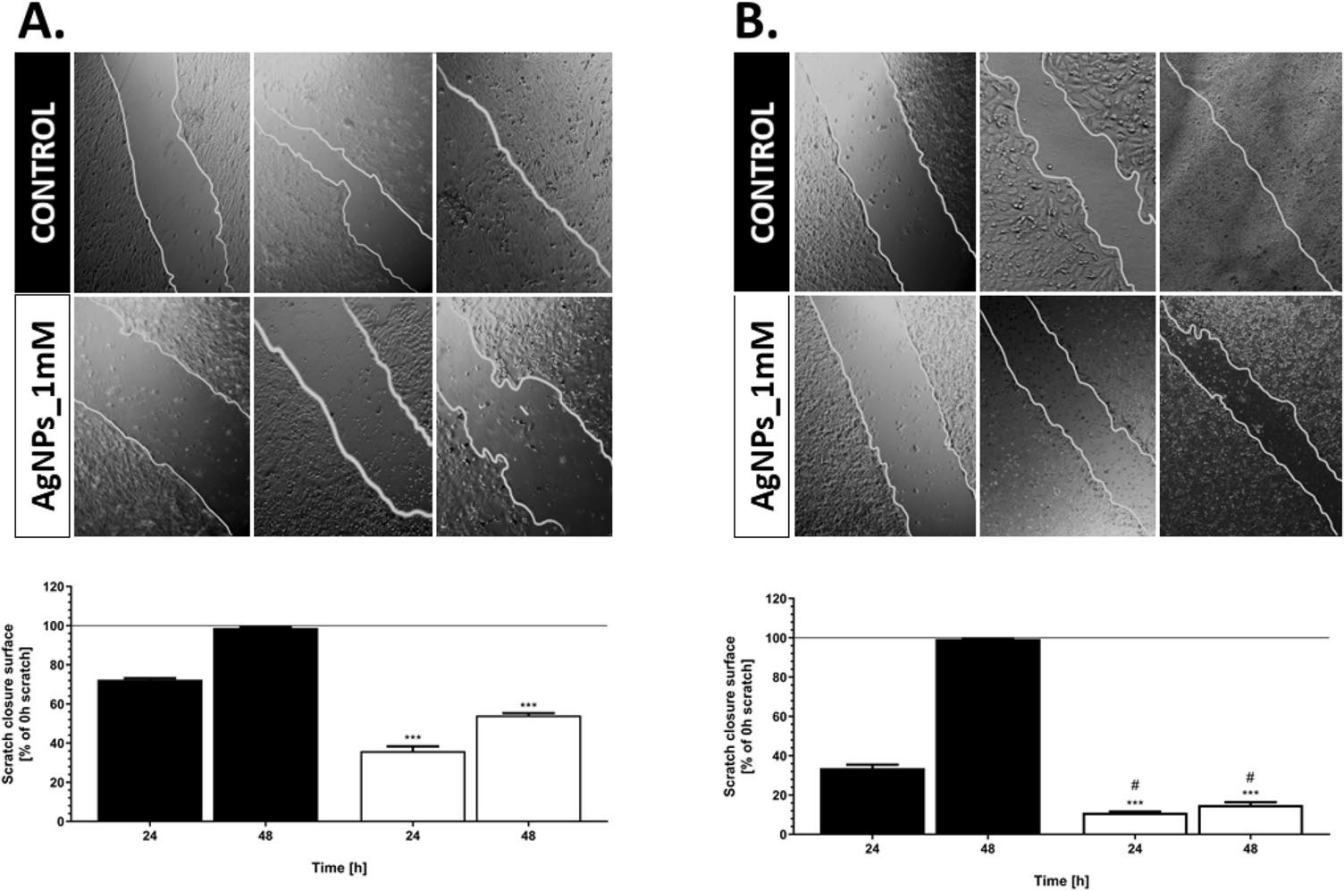
Cell migration ability in response to AgNPs. Comparison of migration in both HaCaT (**A**) and U-2OS (**B**) cell lines by taking images at different time intervals (0, 24, 48 h). Results are represented by marking the scratch with parallel lines and visually displaying the number of cells migrated in to the scratch area. The widths were measured using Image J software, and the data were analyzed using Prism 5.0. The values are the means with standard deviation (error bars). The statistical significance is indicated as follows: *P < 0.05, **P < 0.01, ***P < 0.005 according to one-way ANOVA, Tukey test (compared to respective control), and #*P* < 0.05 are statistically different from respectively tested group (HaCaT *vs.* U-2OS).

Cancer cell migration and invasion play a key role in disease progression^77^. Therefore, we further examine the impact of the silver nanoparticles on the behavior of the cancer and normal cells through the scratch assay. The motility capacities of the cells were performed on human keratinocytes and osteosarcoma cells. After 48 h either HaCaT or U2OS control cells (without AgNPs treatment) were able to migrate and close the scratch (Fig. 9). While, after exposure to AgNPs this ability was inhibited. Although, the migration ability of both of the tested cell line decreased after nanomaterial exposure, there is a difference among each type of cell. AgNPs inhibited more the migratory capability of the human osteosarcoma cell, compared to keratinocytes. After 48 h the % of scratch closure compare to time 0 was equal to 54% and 15% for normal and cancer cells, respectively. Many researchers emphasize on cytotoxicity of the metal NPs and highlight the migration of tumor cell and metastasis-related ability may be impacted by nanomaterials^78^. Herein, the strong inhibition efficacy of AgNPs on migration was observed in cancer cells, which were in line with other group^79,80^. Thus, this suggests that silver nanoparticles may have potential function in the inhibition of the metastasis.

### Penetration of the root canal of the tooth by AgNPs

**Figure 10:**
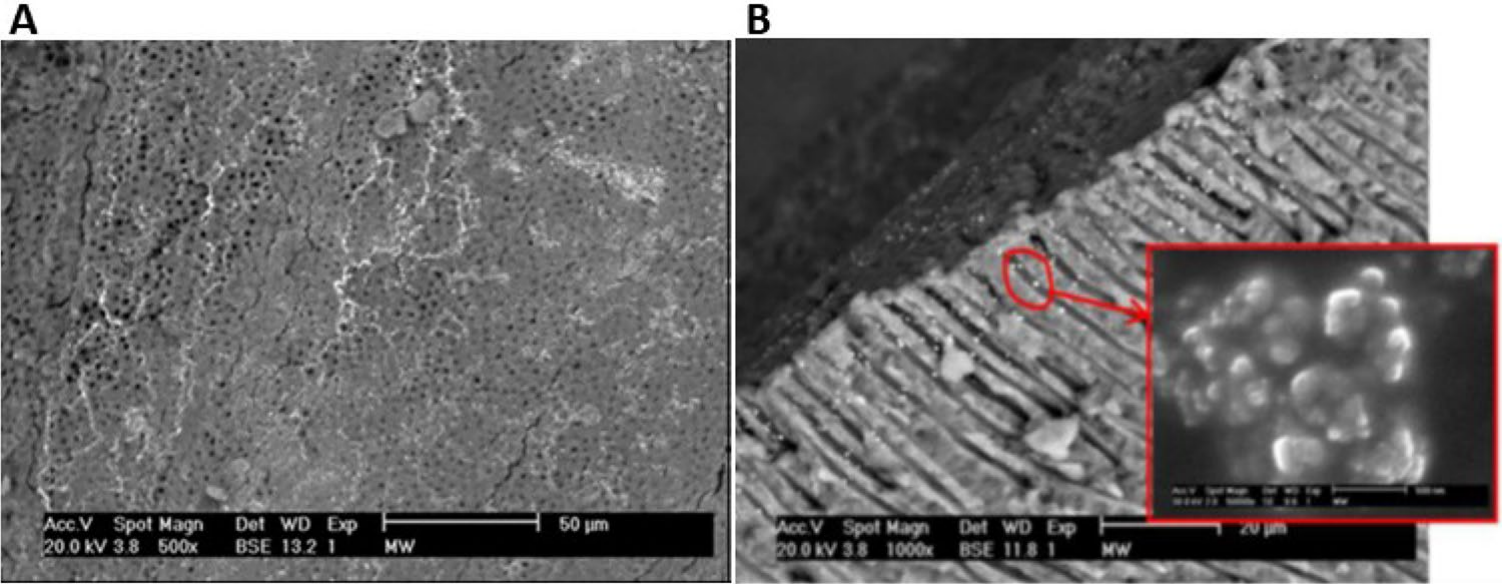
The wall of the macrocanal covered with silver nanoparticles (**A**) and the micrograp of the penetration of silver nanoparticles into the dentinal canal (**B**) with agglomerates of nanoparticles (inset).

As can be seen from the obtained photomicrographs, the walls of the root canal are covered with silver nanoparticles (Fig. 10A). In addition, the penetration of nanoparticles into the dentin structure is observed. The depth of free penetration of silver nanoparticles in the dentinal tubule is about 20 μm (Fig. 10B), which is an extremely important experimental fact^81^. From a physical point of view, the dentin-nanoparticle system tries to reach thermodynamic balance. Nanoparticles tend to occupy a position that corresponds to their minimum energy costs. Such conditions have been found in the developed system of dentinal microcanals, penetrating and lingering in them at a certain depth. Thereby causing a deep bactericidal effect on the pathogenic microflora^82^. We observe that the size of silver inclusions distributed at different depths in the microtubules is preferably slightly larger than the stated 15 nm sizes of nanoparticles (Fig. 10B). This is due to their tendency over time to agglomerations and clustering. This fact can play another, no less important role. Agglomerates of nanoparticles, the size of which acquires the measurement of the diameter of the microtubules, reliably block the return of bacteria from the periphery of the dentin to the macrocanal of the root^43,83^. Thus, the binary action of nanoparticles actually significantly enhances the bactericidal effect of silver.

The green synthesis of silver NPs is usually based on either the whole yeast cells or cell extract. In case of metal nanoparticles are formed with using yeast biomass, they can accumulate inside the cells in response to exposure to metal ions and additional steps are demand to extract the nanomaterials^47,84^. So far, there is lack of the data where the nanoparticles biomanufacturing is performed with the post-culturing water. In that way, the yeast biomass can be easy obtained as the waste product, and may be used many times for preparing the nanomaterials. Moreover, the low-cost of their production will take place in case of culturing in the huge bioreactors at the industrial scale, which does not require the complicated down-stream process for the recovery of the silver nanoparticles. What more, due to antibacterial effect these materials may be useful for various biomedical application, i.e. as antimicrobial and disinfect agent, or to prepare the antiseptic layers^85–87^. Simultaneously, the same nanomaterial, depends on the concentration used, could be a great platform for targeted drug delivery, as well as to combat with cancer cells^88,89^. Altogether, the presented method of AgNPs synthesis, is a simple, cost-effective and efficient approach to obtain the nanomaterials.

## Conclusions

The presented work was focused on the physicochemical and biological characterization of biosynthesized silver nanoparticles. It was found that manufactured AgNPs had spherical shape with the size range between 17–20 nm, depends on the concentration of the silver ions. TEM, DLS and PdI analysis pointed out that AgNPs were administered as a stable and a well dispersed single particles suspension. The EDX map of sulfur located around the nanoparticles suggests that some proteins or sulfur-rich biocompund coat the AgNPs surface and stabilize them. A series of assays with bacteria and fungi strains have confirmed the antimicrobial activity of the AgNPs. Importantly from the potential biomedical application, beside the pronounced antibacterial activity, the reduced cytotoxic effect towards mammalian somatic and tumoral cells was confirmed. Moreover, the ability to deep penetration of the silver colloid to the root canal, imaged by SEM, highlight its potential as the material for root-end filling.
